# Triple Immunoglobulin Gene Knockout Transchromosomic Cattle: Bovine Lambda Cluster Deletion and Its Effect on Fully Human Polyclonal Antibody Production

**DOI:** 10.1371/journal.pone.0090383

**Published:** 2014-03-06

**Authors:** Hiroaki Matsushita, Akiko Sano, Hua Wu, Jin-an Jiao, Poothappillai Kasinathan, Eddie J. Sullivan, Zhongde Wang, Yoshimi Kuroiwa

**Affiliations:** 1 Sanford Applied Biosciences L.L.C., Sioux Falls, South Dakota, United States of America; 2 Kyowa Hakko Kirin, Co., Ltd., Chiyoda-ku, Tokyo, Japan; 3 Department of Animal, Dairy and Veterinary Sciences, Utah State University, Logan, Utah, United States of America; 4 Trans Ova Genetics, Sioux Center, Iowa, United States of America; 5 Hematech, Inc., Sioux Falls, South Dakota, United States of America; Michigan State University, United States of America

## Abstract

Towards the goal of producing fully human polyclonal antibodies (hpAbs or hIgGs) in transchromosomic (Tc) cattle, we previously reported that Tc cattle carrying a human artificial chromosome (HAC) comprising the entire unrearranged human immunoglobulin (Ig) heavy-chain (h*IGH*), kappa-chain (h*IGK*), and lambda-chain (h*IGL*) germline loci produced physiological levels of hIgGs when both of the bovine immunoglobulin mu heavy-chains, b*IGHM* and b*IGHML1*, were homozygously inactivated (b*IGHM^−/−^*, b*IGHML1^−/−^*; double knockouts or DKO). However, because endogenous bovine immunoglobulin light chain loci are still intact, the light chains are produced both from the h*IGK* and h*IGL* genomic loci on the HAC and from the endogenous bovine kappa-chain (b*IGK*) and lambda-chain (b*IGL*) genomic loci, resulting in the production of fully hIgGs (both Ig heavy-chains and light-chains are of human origin: hIgG/hIgκ or hIgG/hIgλ) and chimeric hIgGs (Ig heavy-chains are of human origin while the Ig light-chains are of bovine origin: hIgG/bIgκ or hIgG/bIgλ). To improve fully hIgG production in Tc cattle, we here report the deletion of the entire b*IGL* joining (J) and constant (C) gene cluster (b*IGLJ1-IGLC1* to b*IGLJ5-IGLC5*) by employing Cre/loxP mediated site-specific chromosome recombination and the production of triple knockout (b*IGHM^−/−^*, b*IGHML1^−/−^* and b*IGL^−/−^*; TKO) Tc cattle. We further demonstrate that b*IGL* cluster deletion greatly improves fully hIgGs production in the sera of TKO Tc cattle, with 51.3% fully hIgGs (hIgG/hIgκ plus hIgG/hIgλ).

## Introduction

Human polyclonal antibodies (hpAbs or hIgGs) prepared from plasma donated from the general population or convalescing human donors have been widely used to treat several human diseases, such as autoimmunity, immunodeficiency and infection [1]. In the case of a medical emergency crisis, such as severe acute respiratory syndrome (SARS) outbreaks or the most recent outbreak of Middle Eastern Respiratory Syndrome Coronavirus (MERS-CoV), where no effective treatment is available, hIgGs have been shown to be a life saving measure [2]; Hilgenfeld, R and Peiris, M. 2013). Unfortunately, due to the voluntarily nature of plasma donation, hIgGs and convalescent plasma can be difficult to source.

We previously reported our success in creating double knockout (DKO) transchromosomic (Tc) cattle that produce physiological levels of hIgGs (Sano A *et al.*, 2013). These Tc cattle were engineered by homozygous knockout of both of the bovine immunoglobulin mu heavy-chain genes, b*IGHM* and b*IGHML1* (b*IGHM^−/−^*, b*IGHML1^−/−^*; DKO) and by reconstituting B cell function with a human artificial chromosome (HAC) comprising the entire unrearranged human immunoglobulin heavy-chain (h*IGH*), kappa-chain (h*IGK*), and lambda-chain (h*IGL*) germline loci. However, as the bovine endogenous immunoglobulin light-chain loci, the lambda (b*IGL*) locus and the kappa (b*IGK*) locus, are intact in these Tc cattle, the light-chains are expressed both from h*IGK* and h*IGL* loci on the HAC and from the endogenous b*IGL* and b*IGK* loci, resulting in a mixture of hIgGs composing fully hIgGs (both the heavy-chains and light-chains are of human origin: hIgG/hIgκ and hIgG/hIgλ) and chimeric hIgGs (only the heavy-chains are of human origin but the light-chains are of bovine origin: hIgG/bIgκ and hIgG/bIgλ). Specifically, when the hIgG isotype was analyzed in the sera of Tc bovine, hIgG/bIgλ accounted for about 70% and hIgG/bIgκ accounted for about 10% of the total hIgGs, respectively, while fully hIgG only accounted for about 20% of the total hIgGs (Sano A *et al*., 2013). We reasoned that the human immunoglobulin light-chain genes carried on the HAC are controlled by human regulatory sequences and may be at a disadvantage in competing with the endogenous bovine immunoglobulin light-chain genes for expression in the bovine B cell environment. As bIgλ is the predominantly expressed light-chain isotype in bovine B cells, we further reasoned that inactivation of bovine lambda light-chain genes, in addition to the KO of the two b*IGHM* and b*IGHML1* loci, would dramatically improve fully hIgG production in the resulting Tc cattle.

Here we report our success in employing a Cre/loxP-mediated site-specific chromosome recombination strategy to delete the entire bovine lambda *J* and *C* gene cluster (b*IGLJ1-IGLC1* to b*IGLJ5-IGLC5*) in bovine fibroblast cells. To the best of our knowledge, this is the first report of the successful deletion of an unusually large section of chromosomic DNA (>27 kb) in primary somatic cells with the Cre/loxP system followed by the birth of live healthy offspring, providing a useful protocol in using this system for genome engineering in somatic cells and for transgenic animal production. We here report the cloning of triple KO (TKO, *IGHM^−/−^IGHML1^−/−^IGL^−/−^*) Tc cattle by chromatin transfer (CT). Furthermore, we demonstrate that inactivation of the b*IGL* by deleting the b*IGLJ-IGLC* gene cluster greatly improved fully hIgGs production in the sera of Tc cattle, with an average of 51.3% fully hIgG (hIgG/hIgκ plus hIgG/hIgλ).

## Results

### An animal breeding-assisted sequential gene targeting strategy

**Figure 1:**
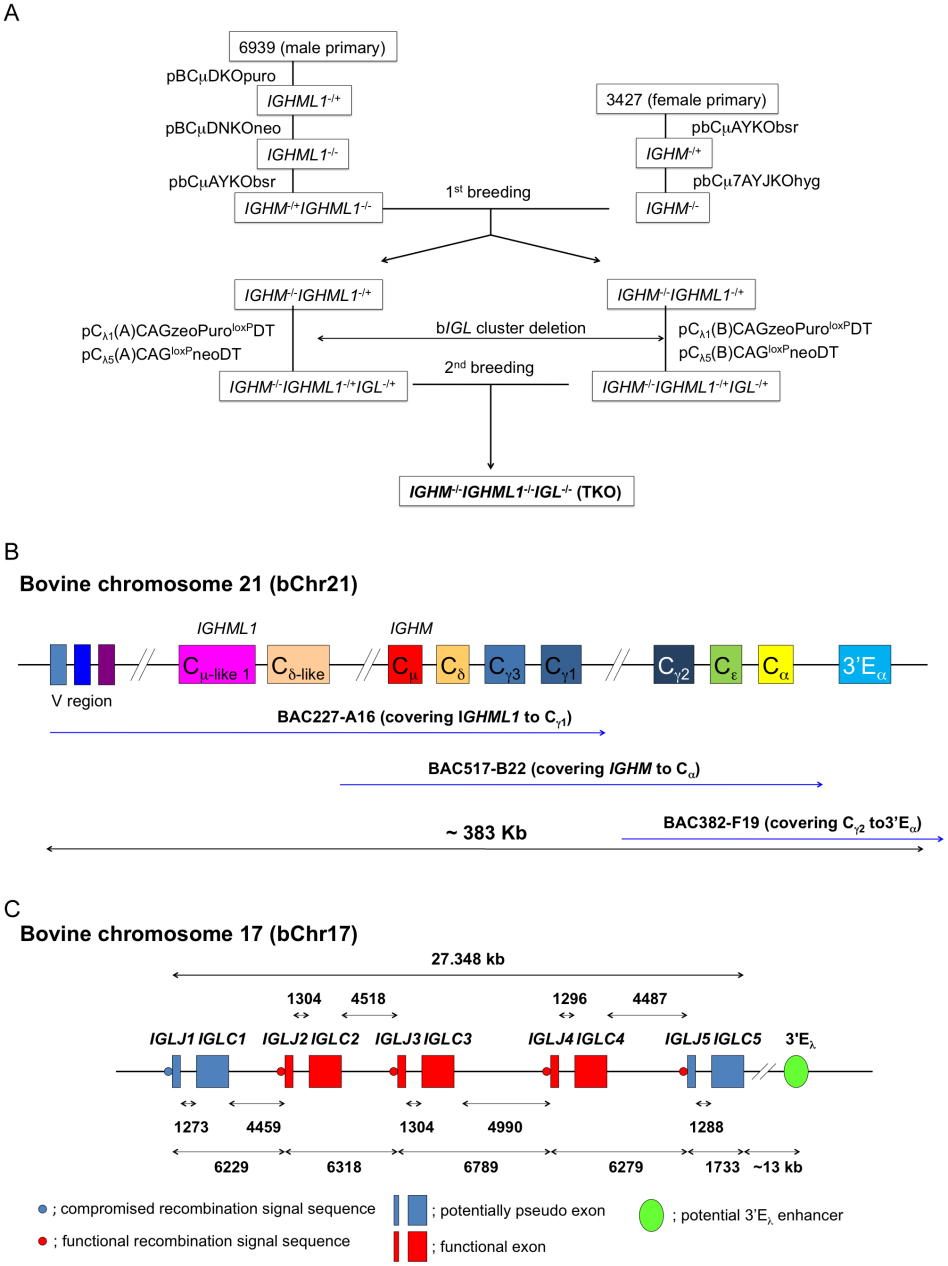
The chromosome engineering strategy including pedigree and deduced structure of bovine chromosomes for knockouts is shown. (A) Breeding pedigree to establish G0 TKO cell lines. Male cell line 6939 and female cell line 3427 were sequentially targeted to obtain *IGHM ^−^* ^/+^ *IGHML1^−^* ^/*−*^ and *IGHM^−^* ^/*−*^ animals, respectively, for the first round of breeding, which generated both male and female G0 *IGHM^−^* ^/*−*^ *IGHML1^−^* ^/+^ cell lines. These cell lines were subjected to the cluster deletion to generate *IGHM^−/−^IGHML1^−/+^IGL^−/+^* animals for the second round of breeding, which led to establishment of male and female G0 TKO cell lines. (B) Deduced structure of the bovine *IGH* gene cluster on bChr21. BAC clone 227-A16 contained part of the *IGH* variable region and the *IGHML1* through the C_γ1_ region. BAC includes the C_γ2_ through the 3′E_α_ region. The size of the three BAC clones contiguously is estimated to be around 380 kb. (C) Genomic organization of the bovine *IGLJ-IGLC* gene cluster on bChr17.

We previously developed a sequential gene targeting strategy and succeeded in producing cloned cattle carrying homozygous knockout of both the immunoglobulin mu and the prion proteins [3]. One of the core components of this sequential gene targeting strategy is to use embryonic cloning by CT to rejuvenate the genetically modified cells following each round of gene targeting [3], [4]. This sequential gene targeting strategy overcomes the limitation set by the cellular senesce program in cultured somatic cells and, in theory, allows one to sequentially modify the genome of somatic cells as many rounds as one wishes. However, as every round of gene targeting requires a round of embryonic cloning by CT to rejuvenate the cells, our recent results showed that cumulative epigenetic errors can be introduced into the (re)cloned cells at each round of embryonic cloning, which in turn severely compromises the developmental competence of the cloned embryos reconstituted from such sequentially (re)cloned cells (our unpublished data). As the goal of this project was to produce TKO Tc cattle where the two b*IGH* loci, *IGHM* and *IGHML*, and the b*IGL* locus, *IGLJ-IGLC* gene cluster, were to be homozygously inactivated, it would take four rounds of gene targeting and embryonic cloning to KO the *IGHM* and *IGHML* loci and another four more rounds to KO the b*IGL* locus (see below for details), entailing eight rounds of gene targeting and embryonic cloning in total. Even though we succeeded in cloning transgenic cattle after seven rounds of embryonic cloning, the cloning efficiency was extremely low [5]. Therefore, we designed a new sequential gene targeting strategy by incorporating animal breeding as an integral component. Specifically, after two to three rounds of gene targeting and embryonic cloning, live animals were produced by CT and raised to sexual maturity for breeding. It is believed, and has been proven by us (our unpublished data), that germline transmission of the cloned genome erases the epigenetic errors acquired from cloning. Therefore, primary cell lines carrying the introduced genetic modifications can be established from the fetuses produced from breeding (we call the cell lines established from fetuses produced by fertilization G0 cell lines) and more rounds of genetic modifications can proceed (i.e. G1, G2…). Figure 1A depicts the gene targeting scheme that we undertook to sequentially KO the b*IGHM, IGHML1* and *IGL* loci for producing TKO cell lines. Of note, contrary to what has been reported by others that the *IGHML1* locus was mapped on bovine chromosome 11 (bChr11) (Hosseini, A *et al*. 2004), our sequence analysis of the bovine *IGHM* and *IGHML1* loci demonstrated that both of these loci are located on bChr21 (our unpublished data). Such chromosomal localization dictates that the *IGHM* and *IGHML1* loci segregate together during meiosis, allowing us to implement the animal breeding-assisted sequential gene targeting strategy and calculate the probability of the desired genotypes of fetuses produced by breeding (hence the cell lines established from the fetuses) of these two genomic loci as depicted in Figure 1B.

### Sequential gene targeting of bovine *IGHM* and *IGHML1* and establishment of male and female *IGHM^−^* ^/*−*^ *IGHML1^−^* ^/+^cell lines

For production of breeding cattle carrying the desired genetic modifications in the b*IGH* loci as depicted in Figure 1A, we targeted the b*IGH* loci in both male and female bovine cell lines by employing the sequential gene targeting strategy that we developed previously [3]. For males, we selected a Holstein fetal primary fibroblast cell line (6939) and sequentially knocked out both of the *IGHML1* alleles (we designated the two alleles of the *IGHML1* locus in this cell line as *U* and *u*, respectively) and *AY* allele of the *IGHM* locus (we designated the two alleles of the *IGHM* locus in this cell line as *AY* and *ay*, respectively) and established *IGHM^−^* ^/+^ *IGHML1^−^* ^/*−*^ cell lines. The sequential gene targeting for each of the gene alleles was achieved using different gene targeting vectors carrying different drug selection markers, bCµAYKObsr for *AY*, pBCµΔNKOneo for *U*, and pBCμΔKOpuro for *u* as reported previously [5]. As these cell lines underwent three rounds of embryonic cloning by CT without going through germline transmission by breeding, we designate them generation 3 (G3) cell lines.

For females, we used a primary fetal fibroblast cell line (3427) established from a Holstein x Jersey cross (HoJo) and sequentially knocked out both of the *IGHM* alleles (we designated the two *IGHM* alleles as *10AY* and *7AYJ*, respectively, and the two alleles of *IGHML1* as 8*U* and 5*u*, respectively, in this cell line) with gene targeting vectors pbCμAYKObsr for *10AY* and pbCμ7AYJKOhyg for *7AYJ*, respectively (Figure 1A) leading to the established *IGHM^−^* ^/*−*^ cell lines. As defined above, these are generation 2 (G2) cell lines.

We then produced calves from both the male *IGHM^−^* ^/+^ *IGHML1^−^* ^/*−*^ and female *IGHM^−^* ^/*−*^ cell lines by CT (Table 1). As one of the *IGHM* alleles in the males and two of *IGHML1* alleles in the females were intact, the resulting animals were immunologically competent, allowing them to survive and breed without complications associated with being immune compromised. To produce fetuses for establishing G0 cell lines with the desired genotypes for subsequent bovine b*IGLJ-IGLC* gene cluster deletion, these calves were bred using superovulation followed by artificial insemination and embryo flush/transfer when they were at 18–20 months of age and fetuses were collected at 40 days (40d) of gestation. In total, 18 fetuses were collected and genotyped. Among them, seven fetuses (7/18, 39%) were confirmed to be the *IGHM^−^* ^/*−*^ *IGHML1^−^* ^/+^ genotype, with the rest being the *IGHM^−^* ^/+^ *IGHML1^−^* ^/+^ genotype, a pattern slightly varying from predicted Mendelian inheritance. In all of these seven fetuses, the *neo* KO cassette at *U* and the *bsr* KO cassette at *AY* segregated together, consistent with our sequence analysis that both of the *IGHM* and *IGHML1* loci are located on the bChr21.

**Table 1:** *IGHML^−/−^IGHM^−/+^* (male) and *IGHM^−/−^* (female) calving efficiencies.

Modification	Recipients Implanted	Pregnant at 40d (%)	Fetus collected at 40d	150-180d	Live Calves born (%)	Calf ID used for breeding
*IGHML−/− IGHM−/+* Male	65	59 (91)	12 (19)	27 (42)	12 (18)	305
*IGHM−/−* Female	62	34 (55)	4 (6)	20 (32)	15 (24)	1295 1296 1305

### Characterization of the b*IGLJ-IGLC* gene cluster in the b*IGL* locus

As there was insufficient information about the sequence and gene structures of the *IGLJ-IGLC* gene cluster in the bovine genome [6], [7] to design and construct gene-targeting vectors, we first isolated and characterized a bovine bacterial artificial chromosome (BAC) DNA sequence from this gene cluster. By screening a BAC genomic library with probes specific to the bovine immunoglobulin lambda constant region sequences (GenBank AF396698); we isolated a positive BAC clone and subjected it to shotgun sequencing. Sequence analysis (Figure S3) led to the identification of the entire bovine *IGLJ-IGLC* gene cluster composed of the five *IGLJ*-*IGLC* genes (*IGLJ1-IGLC1* through *IGLJ5-IGLC5*) (Figure 1C). Among them, *IGLJ2-IGLC2, IGLJ3-IGLC3* and *IGLJ4-IGLC4* appeared to be functional genes as judged by their uninterrupted reading frames coding for functional proteins (Figure 1C). On the other hand, both the *IGLJ1-IGLC1* and *IGLJ5-IGLC5* genes contain immature stop codon mutations, indicating that they are pseudo genes. We also identified a potential enhancer element, 3′Eλ, about 13 kb downstream of the *IGLJ5-IGLC5* gene that shares ∼60% DNA sequence homology with the human 3′Eλ (HSS-3) enhancer sequence. With the identification of this 3′Eλ enhancer 13 kb downstream of the *IGLJ5-IGLC5* gene on the same BAC clone, we concluded that the *IGLJ5-IGLC5* gene is the last gene unit in the bovine *IGLJ-IGLC* gene cluster.

**Figure 2:**
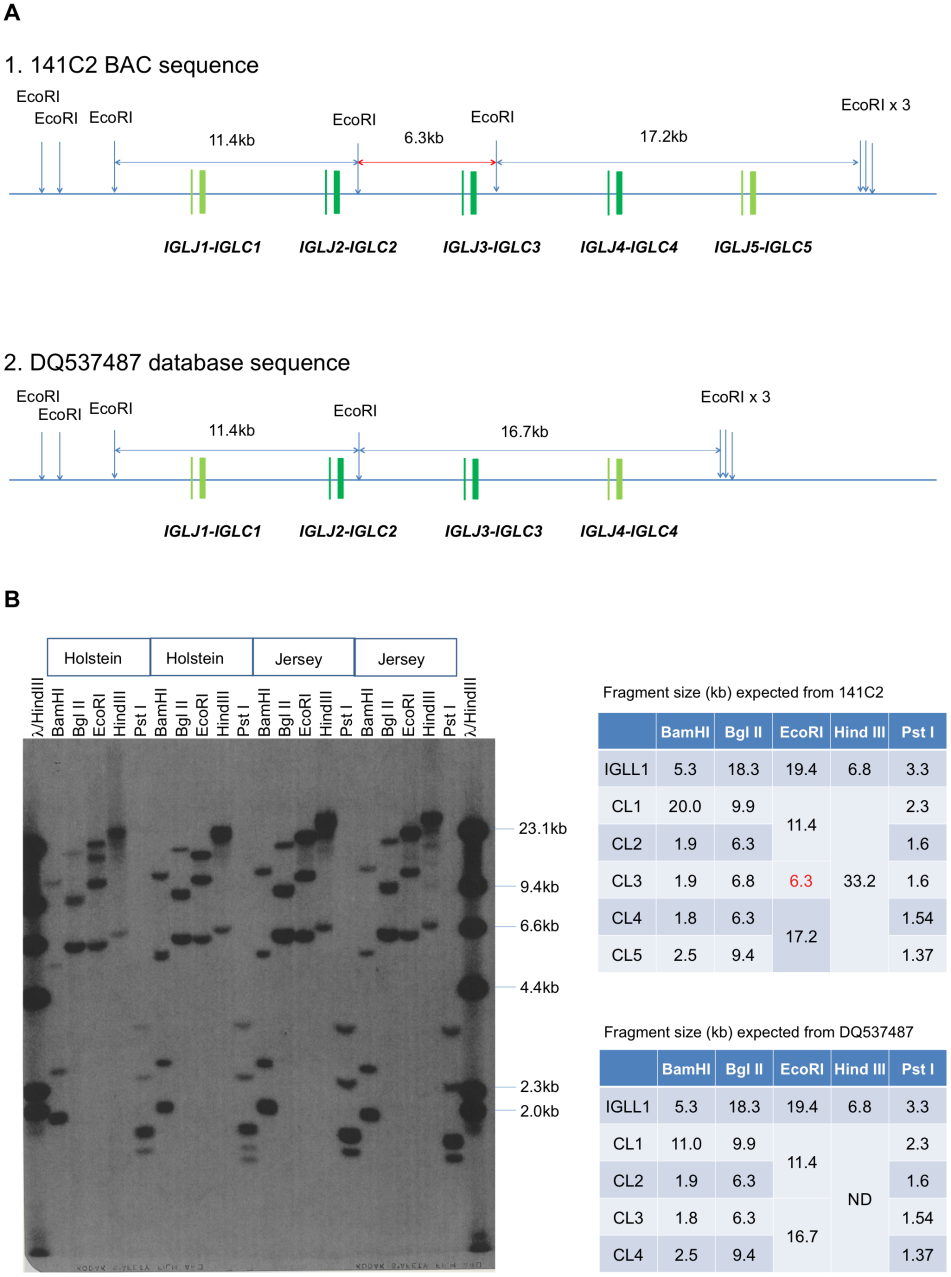
Southern blot analysis of bovine genomic DNA for the analysis of *IGLJ-IGLC* gene cluster including deduced restriction fragments is shown. (A) Deduced size of EcoRI fragments based on the sequence data of 141C2 and DQ587487. The most significant difference in the fragment is the presence or absence of 6.3 kb EcoRI fragment stained with probe that was prepared based on bovine *IGLC* sequence(GenBank AF396698). (B) Southern blot results of two Holstein and two Jersey genomic samples digested with BamHI, Bgl II, EcoRI, Hind III, and Pst I stained with the probe that was prepared based on bovine *IGLC* sequence (GenBank AF396698). The expected sizes of fragments are listed in the upper and lower table based on the 141C2 and DQ587487 sequences, respectively.

Compared to the report by Chen, L *et al.* where four *IGLJ-IGLC* genes were identified in the bovine [6], we identified five *IGLJ-IGLC* genes based on our sequence data. We found that there is a duplication of the originally identified *IGLJ2-IGLC2* by Chen *et al.* and that the duplicated genes are identical in their exon sequences but are subtly different in their intron and 3′ untranslated region (UTR) sequences. As such, we concluded that this is a distinct *IGLJ-IGLC* gene from *IGLJ2-IGLC2* and defined it as *IGLJ3-IGLC3*. Our sequence analysis also indicates that this gene is located about 4 kb downstream of *IGLJ2-IGLC2* (Figure 1C). The discrepancy on the numbers of *IGLJ* gene unites in the b*IGL* locus between what was found by Chen *et al*. and by us is likely attributed to the fact that the coding sequences of *IGLJ2-IGLC2* and *IGLJ3-IGLC3* exons are identical and were not detected as distinct gene units by Chen L *et al*. However, our detailed sequence analysis showed that these two gene units are different in the sequences of introns and 3′ UTRs (Figure S1 Panels A and B). Our interpretation of the structure of the *IGLJ-IGLC* gene cluster was further supported by Southern blot data we produced from analyzing the genomic DNA isolated from fibroblast cells derived both from Holstein and Jersey breed cattle (Figure 2). Due to the relatively short shotgun sequences (1.5–3.0 kb by Chen *et al*.), genes with high sequence homologies, such as between *IGLJ2-IGLC2* and *IGLJ3-IGLC3,* may not be readily identified as distinct genes if the sequence assembled from shotgun sequencing is the only resource used for characterizing a genomic locus. Therefore, our Southern blot data provided unequivocal support for our conclusion that there are five *IGLJ*-*IGLC* genes (*IGLJ1-IGLC1* through *IGLJ5-IGLC5*) in the bovine genome.

### Deletion of b*IGLJ-IGLC* gene cluster through Cre/loxP mediated site-specific chromosomal recombination and establishment of male and female G0 *IGHM* ^−/−^ *IGHML1* ^−/−^ *IGL* ^−/−^ cell lines

**Figure 3:**
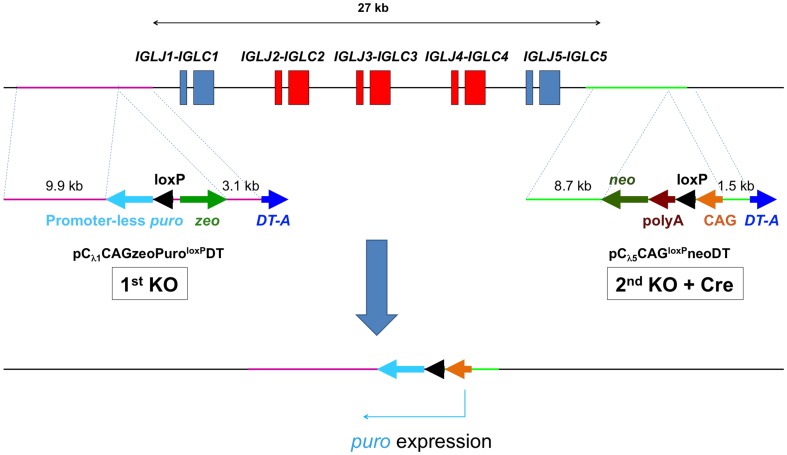
Strategy for the bovine lambda cluster deletion is shown. The plasmid pC_λ1_CAGzeoPuro^loxP^DT is the first KO vector to place a loxP site 5′ outside of the J_λ1_ domain, composed of 9.9 kb and 3.1 kb genomic DNA as a long and short arm, respectively, a loxP sequence, CAG promoter-driven zeocin-resistant gene, DT-A and promoter-less puromycin-resistant gene. The plasmid pC_λ5_CAG^loxP^neoDT is a second KO vector allowing placement of another loxP site 3′ outside of C_λ5_ domain, composed of 8.7 kb and 1.5 kb genomic DNA as a long and short arm, respectively, a loxP sequence, chicken β-actin promoter-driven neomycin-resistant gene, DT-A, SV40 polyA and CAG promoter. The second KO vector is co-transfected with Cre-expression plasmid to bring about the cluster deletion, which reconstitutes CAG promoter-driven puromycin-resistant gene. Therefore, cells where the cluster deletion occurred can be selected by puromycin.

Since the b*IGL* locus contains multiple functional *IGLJ-IGLC* gene units as described above, we decided to fully inactivate the bovine lambda genes by deleting the entire *IGLJ-IGLC* gene cluster with Cre/loxP-mediated chromosomal recombination. To this end, we designed and constructed knock in (KI) vectors to integrate loxP sequences, one at 129 bp 5′ of the b*IGLJ-IGLC1* gene and another at 295 bp 3′ of *IGLJ5-IGLC5* gene, through homologous recombination to flank the entire b*IGLJ-IGLC* gene cluster (Figure 3). For aiding the identification of cell clones in which the correct b*IGLJ-IGLC* cluster deletion occurred, we designed the two loxP KI vectors in such a way that the promoterless puromycin gene (*puro*) introduced by the KI vector pC_λ1_CAGzeoPuro^loxP^DT would be activated by the CAG promoter introduced by the KI vector pC_λ5_CAG^loxP^neoDT when the Cre-mediated DNA recombination occurred between the two loxP sites introduced by these two KI vectors (Figure 3).

**Figure 4:**
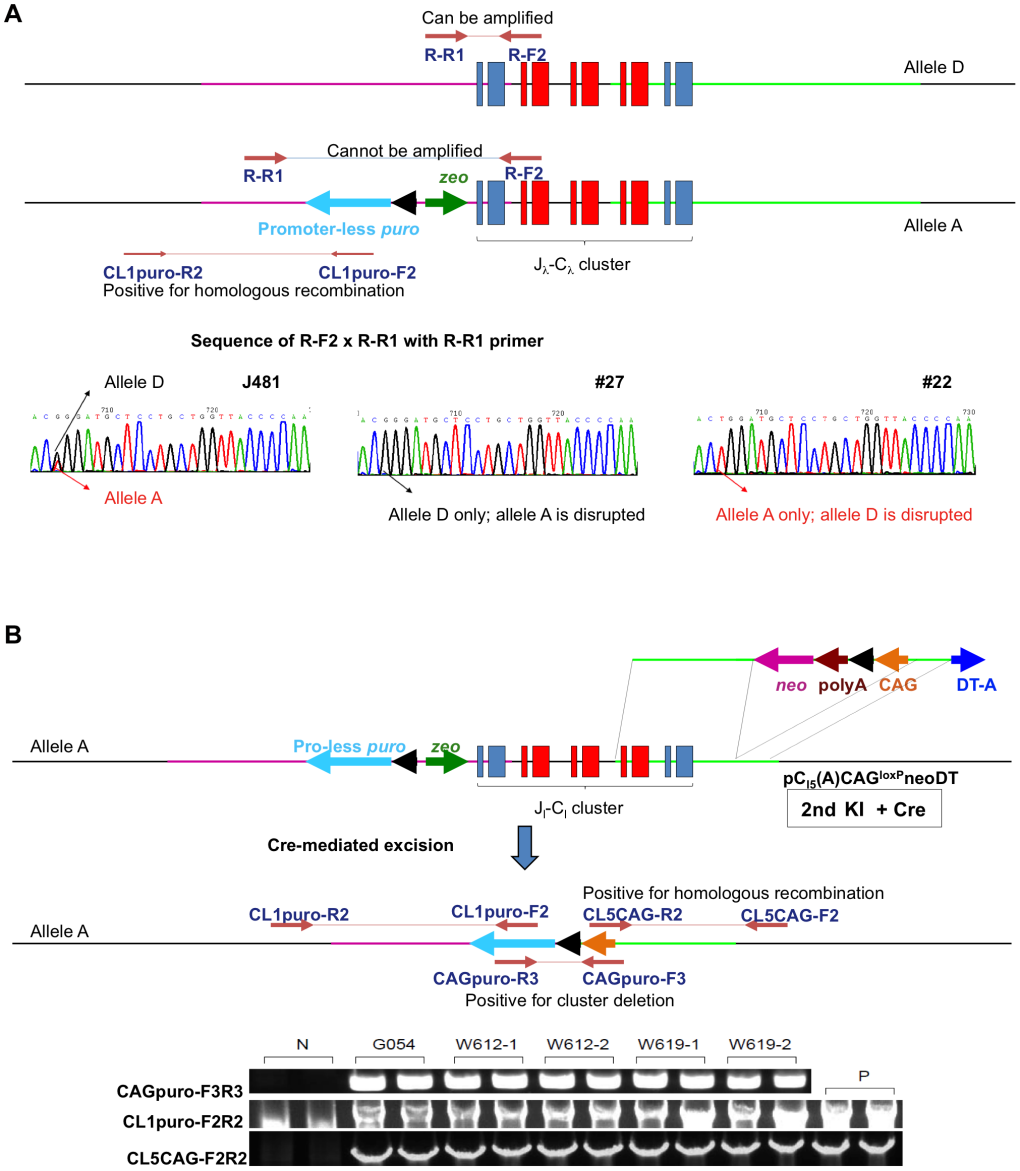
Integration of two loxP sequences to enable cluster deletion is shown. (A) Integration of a loxP sequence 5′ outside of the J_λ1_ domain by the targeting vector pC_λ1_CAGzeoPuro^loxP^DT in cell line J481. The occurrence of homologous recombination was confirmed by genomic PCR, CL1puro-F2F2, as positive PCR. Furthermore, negative PCR, R-F2 x R-R1, was done to double check the homologous recombination because it can only be amplified from the wild type allele; J481 amplified this sequence from both alleles *A* and *D*, showing the double peaks (T for allele *A* and G for allele *D*). Colony #27 showed only “G”, demonstrating that allele *A* was specifically knocked out while Colony #22 showed only “T”, demonstrating that allele *D* was specifically knocked out. From colony #27, fetal cell line, K655-1, was established, which was positive with CL1puro-F2R2 primers. (B) Integration of another loxP sequence located 3′ outside the C_λ5_ domain by the targeting vector pC_λ5_(A)CAG^loxP^neoDT in cell line K655-1. The occurrence of homologous recombination was confirmed by genomic PCR, CL5CAG-F2F2, as positive PCR. Moreover, the reconstitution of CAG promoter-driven puro gene caused by the cluster deletion was also confirmed by genomic PCR, CAGpuro-F3R3. Cell line G054 was used to generate calves for breeding.

We conducted b*IGLJ-IGLC* gene cluster deletions both in a male cell line, J481 (*IGHM* ^−/−^ *IGHML1* ^−/+^), and a female cell line, H412 (*IGHM* ^−/−^ *IGHML1* ^−/+^), which were established above. For distinguishing the b*IGL* alleles in these two cell lines where single nucleotide polymorphisms (SNPs) were identified (Figure 4) and for designing and constructing allele-specific KI vectors, we designated the b*IGL* alleles in J481 as *A* and *D*, and those in H412 as *B* and *C*, respectively (Figure 4A; only *A* and *D* alleles in J481 are depicted). To delete the b*IGLJ-IGLC* gene cluster in the J481 cell line, we first transfected J481 cells with the allele *A-*specific KI vector, pC_λ1_(A)CAGzeoPuro^loxP^DT, to integrate the loxP sequence at a position 5′ of the b*IGLJ-IGLC1* gene. After selection of transfected cells with zeocin, the zeocin-resistant cell colonies were screened for the desired gene targeting event with genomic PCR. As depicted in (Figure 4A), we designed a pair of PCR primers, CL1puro-F2/CL1puro-R2, that specifically amplified the DNA sequence generated by the correct integration of the loxP sequence through homologous recombination at a position 5′ of the b*IGLJ1-IGLC1* gene. Following sequencing of the PCR products, 18% (25/140) of the zeocin-resistant colonies were confirmed to be correctly targeted. We also used another pair of PCR primers, R-R1 x R-F2, flanking the SNPs that distinguished the *A* and *D* alleles and specifically amplified the untargeted allele to investigate which of the two alleles were targeted. By sequencing the PCR products generated from this pair of primers, we identified a cell colony, #27, that was positive for the nucleotide G (specific to allele *D*) but negative for nucleotide T (specific to allele *A*) in the SNPs region, indicating that allele *A* was correctly targeted while allele *D* was intact (Figure 4A). We then subjected colony #27 to embryonic cloning to generate 40d fetuses from which five fibroblast cell lines were established. As expected, all five cell lines were confirmed to have the genotype *IGHM* ^−/−^ *IGHML1* ^−/+^ *IGLC1* ^loxP^. We chose one cell line, K655-1, to conduct subsequent gene targeting experiments.

To integrate the second loxP sequence at a position 3′ of the b*IGLJ5-IGLC5* gene and to subsequently induce chromosomal recombination between these two integrated loxP sequences to delete the b*IGLJ-IGLC* gene cluster via Cre-expression, we tested whether co-transfection of K655-1 cells with the second loxP KI vector and Cre-expression plasmids together was effective to achieve this goal. We performed the experiments with the intention to reduce one round of transfection and one round of embryonic cloning in deleting the b*IGLJ-IGLC* gene cluster; otherwise, one round of transfection with the second loxP KI vector followed by one round of embryonic cloning would be needed before transfecting the cells with Cre-expression plasmid. After co-transfecting K655-1 cells with the pC_λ5_(A)CAG^loxP^neoDT KI vector designed to be specific to allele *A* and the Cre-expression plasmid, 21 puromycin-resistant colonies were obtained. We subjected these colonies to genomic PCR screening by using two pairs of PCR primers, CL5CAG-F2/CL5CAG-R2 and CAGpuro-F3/CAGpuro-R3, with the former to identify the correct targeting event 3′ of the b*IGLC5* gene and the latter to detect the desired cluster deletion event induced by loxP/Cre-mediated chromosome recombination (Figure 4B). All of the 21 colonies were confirmed to carry the desired b*IGLJ-IGLC* gene cluster deletion by PCR and subsequent sequencing of the PCR products. We then conducted embryonic cloning by using the cells from some of these positive colonies as chromatin donors and established multiple 40d cell lines (G2). As expected, genotyping analysis showed that all of these cell lines carried the b*IGLJ-IGLC* gene cluster deletion as well as the inactivated alleles in the *IGHM* and *IGHML1* loci introduced earlier, confirming the establishment of cell lines with the *IGHM* ^−/−^ *IGHML1* ^−/+^ *IGL* ^−/+^ genotype. We chose cell lines G054 and W612-1, based on preferable epigenetic profiles revealed by a series of epigenetic screening assays that we developed (our unpublished data), as chromatin donors and conducted CT to produce *IGHM* ^−/−^ *IGHML1* ^−/+^ *IGL* ^−/+^genotype male calves. We performed embryo transfer and transferred 69 cloned embryos to recipient cows. Twenty-seven pregnancies were detected at 40d, and eight live calves were produced (Table 2). Male calves 522 from G054 and 526 and 528 from W612-1 were established for the second round of breeding.

**Table 2:** *IGHML* ^−/+^ *IGHM* ^−/−^ *IGL* ^−/+^ calving efficiencies.

Male Fetal Cell line ID	Recipients implanted	Pregnant 40d (%)	Fetus collected at 40d	Pregnant 180d (%)	Live Calves born (%)	Calf ID used for breeding
G054	39	16 (41)	0	9 (23)	4 (10)	522
W612-1	30	11 (37)	0	10 (33)	4 (13)	526, 528

Female Fetal Cell line ID	Recipients implanted	Pregnant 40d (%)	Fetus collected at 40d	Pregnant 180d (%)	Live Calves born (%)	Calf ID used for breeding
M853-2	100	48 (48)	0	14 (14)	7 (7)	1768
Y927-1	31	16 (52)	0	6 (19)	4(13)	1820
Directly from *IGLC* cluster deleted colonies from H375-2	40	22(55)	3 (8)	12(30)	5(13)	1804 1805 1806

In parallel, we employed the same two-step b*IGLJ-IGLC* gene cluster deletion strategy described above in the female cell line H412 (*IGHM* ^−/−^ *IGHML1* ^−/+^). Briefly, we transfected female H412 cells with the allele *B-*specific KI vector, pC_λ1_(B)CAGzeoPuro^loxP^DT, to integrate the loxP sequence at a position 5′ of the b*IGLJ-IGLC1* gene. By screening the resultant zeocin-resistant colonies for the desired targeting event with genomic PCR CL1puro-F2/CL1puro-R2 and sequencing the PCR products, 16 of 202 (7.9%) of the screened zeocin-resistant colonies were confirmed to be correctly targeted. Similar to the experiments carried out in the male cell line, we confirmed allele-specific gene targeting by a pair of PCR primers (R-R1 x R-F2) which flank the SNPs distinguishing the *B* and *C* alleles and specifically amplifies the untargeted allele. We identified five colonies targeted to the *B*-allele as they were positive for nucleotide G (specific to allele *C*) but negative for nucleotide T (specific to allele *B*) in the SNPs region. We then subjected one colony, #97, to embryonic cloning by CT to generate 40d fetuses. We established five fibroblast cell lines from these fetuses and all of them were confirmed to be the *IGHM* ^−/−^ *IGHML1* ^−/+^ *IGLJC1* ^loxP/+^ genotype. We chose one cell line, H375-2, for subsequent gene targeting experiments.

Again, we employed the same co-transfection strategy used in the male cell line to integrate the second loxP sequence at a position 3′ of the b*IGLJ5-IGLC5* gene and to subsequently induce chromosomal recombination between these two integrated loxP sequences to delete the b*IGLJ-IGLC* gene cluster via Cre-expression in the female cell line. In this case, we co-transfected female H375-2 cell line with the pC_λ5_(B)CAG^loxP^neoDT KI vector, designed to be specific to allele *B,* and the Cre-expression plasmid and obtained 15 puromycin-resistant colonies. Genomic PCR screening was conducted using two pairs of PCR primers, CL5CAG-F2/CL5CAG-R2 and CAGpuro-F3/CAGpuro-R3 followed by sequencing of the PCR products which led to the identification of 6 colonies carrying the desired b*IGLJ-IGLC* gene cluster deletion. We subsequently established multiple 40d cell lines (G2) from some of these positive colonies by embryonic cloning. We confirmed the *IGHM* ^−/−^ *IGHML1* ^−/+^ *IGL* ^−/+^ genotype by genotype analysis as conducted in the male cell lines. We chose one cell line, Y927-1, conducted CT and produced *IGHM* ^−/−^ *IGHML1* ^−/+^ *IGL* ^−/+^ female calves. We performed embryo transfer and transferred 171 cloned embryos to recipient cows. Eighty-six pregnancies were detected at 40d, and 16 live calves were produced (Table 2). Female calves 1804, 1805 and 1806 were also produced directly from cluster deleted colonies (Table 2).

For the establishment of female *IGHM* ^−/−^ *IGHML1* ^−/+^ *IGL* ^−/+^ calves, we employed an additional round of cluster deletion using another female *IGHM* ^−/−^ *IGHML1* ^−/+^ cell line, J746. First, we inserted the loxP sequence at 3′ of *IGLJ5-IGLC5* using pC_λ5_(B)CAG^loxP^neoDT and established an *IGHM* ^−/−^ *IGHML1* ^−/+^ *IGLJC1* ^loxP/+^ fetal cell line. Secondly, we inserted the loxP sequence 5′ of *IGLJ1-IGLC1* in cell line J746 using pC_λ1_(B)CAGzeoPuro^loxP^DT, resulting in the establishment of *IGHM* ^−/−^ *IGHML1* ^−/+^ *IGL* ^−/+^ fetal cell line M853-2. From this cell line, female *IGHM* ^−/−^ *IGHML1* ^−/+^ *IGL* ^−/+^ calf 1768 was established through CT (Table 2).

Male (522, 526 and 528) and female (1768, 1804, 1805, 1806 and 1820) cattle were raised to sexual maturity and crossbred (by artificially inseminating the *IGHM* ^−/−^ *IGHML1* ^−/+^ *IGL* ^−/+^ heifers with semen collected from with the *IGHM* ^−/−^ *IGHML1* ^−/+^ *IGL* ^−/+^bulls) when they were at 18–20 months of age. At 40d gestation, 58 fetuses were collected and genotyped. Among them, five fetuses (8.62%) were identified as TKO (*IGHM* ^−/−^ *IGHML1* ^−/−^ *IGL* ^−/−^) and were used to establish fibroblast cell lines (Figure S2). Two of these fetuses were identified as female and three as male using Y-chromosome-specific genomic PCR.

### Production of TKO Tc cattle by chromatin transfer

For improving the level of hIgG production in Tc cattle, we previously engineered a new version of HAC (named cKSL-HACΔ) and have demonstrated that cKSL-HACΔ/DKO Tc cattle produce physiological levels of hIgG (Sano *et al.,* 2013). In comparison to kHAC that we reported previously where only the h*IGH* locus and h*IGK* locus were incorporated [5], all of the human immunoglobulin germline loci, the h*IGH* locus from human chromosome 14 (hChr14), the h*IGK* locus from hChr2, and the surrogate light-chain (h*SLC*) and h*IGL* loci from hChr22 were incorporated (Sano *et al.,* 2013). In addition, in the newly engineered cKSL-HACΔ, the hIgM constant domain which is believed to be involved in the interaction between IgM and transmembrane α (Igα) and β immunoglobulins (Igβ) in the pre-B cell receptor (pre-BCR) complex was replaced by the bovine IgM constant domain, an effort for improving the interactions between hIgM and bovine Igα and Igβ. In addition to the improved levels of hIgG production, this new version of HAC also dramatically improved B cell development in the DKO Tc cattle (Sano *et al.,* 2013). Therefore, we transferred the cKSL-HACΔ from a Chinese Hamster Ovary (CHO) master cell bank that we established (Sano *et al.,* 2013) into the above established TKO bovine fibroblast cells by Micro-cell Mediated Chromosome Transfer (MMCT) and established cKSL-HACΔ/TKO fibroblast cell lines (see Materials and Methods section for details) for TKO Tc cattle production. At the same time, the majority of pregnancy at 40d were maintained (126 out of 151) and 37 live Tc calves were born (Table 3).

**Table 3:** *IGHML* ^−/−^ *IGHM* ^−/−^ *IGL* ^−/−^ cKSL-HACΔ calving efficiencies.

TKO Cell line ID	Recipients implanted	Pregnant 40d (%)	Fetus collected at 40d	Preg 180d (%)	Live Calves born (%)	Established cKSL-HACΔ/TKO Fetal Cell Lines (Evaluated)
A332A	86	27(31)	5 (6)	8(9)	2(2)	
A596A-1	82	17(21)	3 (4)	11(13)	7(9)	R788
E024A-2	221	76(34)	11 (5)	33(15)	21(10)	V524
A114A	48	21(44)	4 (8)	12(25)	6(13)	
C970A	48	10(21)	2 (4)	3(6)	1(2)	

Fetal Cell line ID	Recipients implanted	Pregnant 40d (%)	Pregnant 180d (%)	Live Calves born (%)
R788	32	13(41)	7(22)	5(16)
V524	32	11(34)	7(22)	5(16)

**Figure 5:**
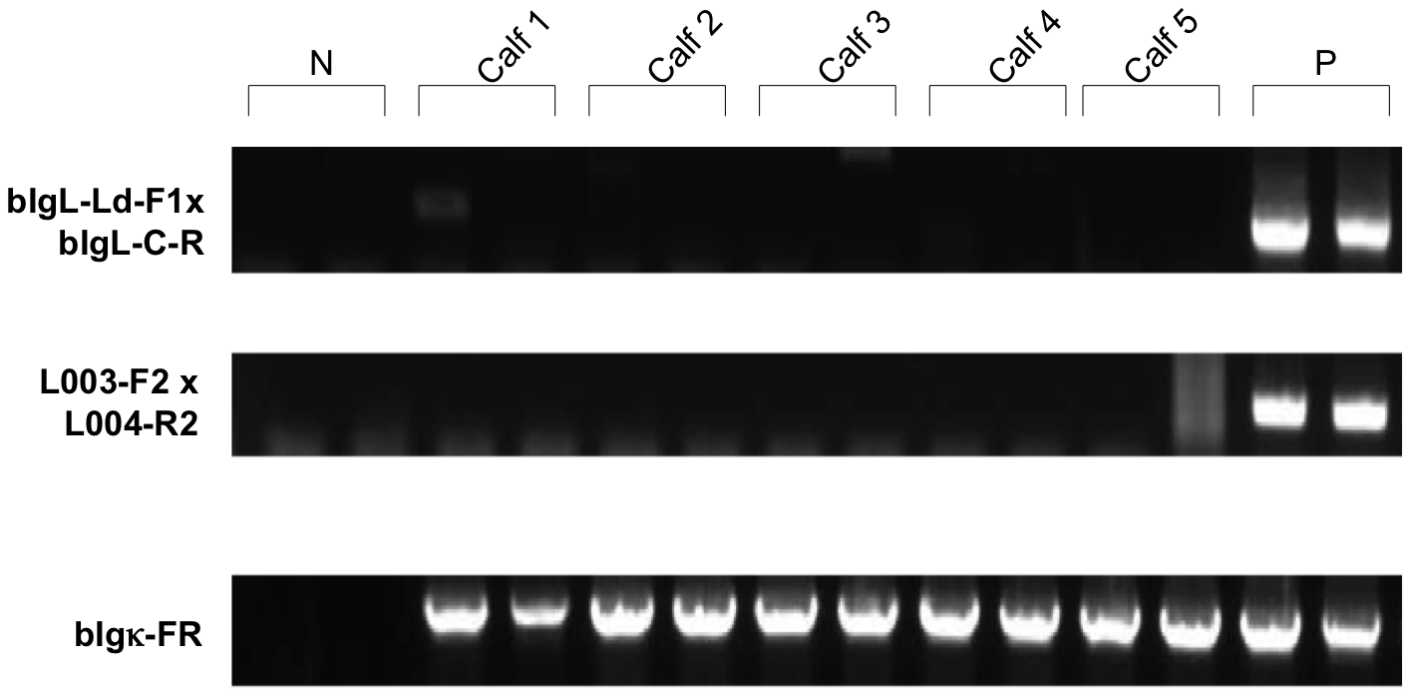
Lack of b*IGL* expression in HAC/TKO calves by RT-PCR is shown. PBMCs from five HAC/TKO calves (Calf 1-5) at birth were subjected to RT-PCR to confirm lack of b*IGL* expression. The primer pairs, bIgL-Ld-F1x bIgL-C-R and L003-F2 x L004-R2, were used to amplify VJ-rearranged b*IGL* and constant region *IGLC* (bC_λ_) genes, respectively. The primer pair, bIgκ-FR, was used to amplify VJ-rearranged b*IGK* gene. N, negative control; P, positive control.

To establish the master cKSL-HACΔ/TKO fetal fibroblast cell banks for continued Tc bovine production, we also retrieved fetuses at 40d from two pregnancies established from the cloning experiments discussed above and established two fetal cell lines, R788 (from a fetus cloned from a cell colony derived from transferring cKSL-HACΔ from CHO cells to the TKO cell line A596A-1) and V524 (from a fetus cloned from a cell colony derived from transferring cKSL-HACΔ from CHO cells to the TKO cell line E024A-2). To test these two newly established cell lines and to clone more Tc calves, we cloned Tc cattle using the two established cKSL-HACΔ/TKO fibroblast fetal cell lines (R788 and V524) as chromatin donors by CT. In total, embryos were transferred to 32 recipient cows for each cell lines and five live calves each were produced (Table 3). RT-PCR analysis on blood cells isolated from the newborn calves demonstrated that deletion of the b*IGLJ-IGLC* gene cluster resulted in a complete elimination of bovine lambda light-chain gene expression (Figure 5).

### Comparison of B cell development and fully hIgG production between cKSL-HACΔ/DKO and cKSL-HACΔ/TKO Tc cattle

**Figure 6:**
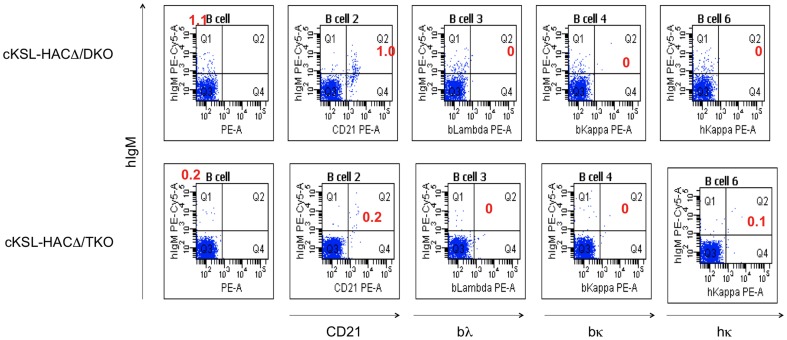
Representative flow cytometry analysis of PBMCs from a series of cKSL-HACΔ/DKO and cKSL-HACΔ/TKO calves at birth is shown. For IgM detection, anti-hIgM antibody was used. From left to right panels, PBMCs were stained with IgM alone, IgM/bCD21, IgM/bλ, IgM/bκ and IgM/hκ antibodies. Each red number represents % of cells in Q1 (IgM alone) or Q2 (IgM/bCD21, IgM/bλ, IgM/bκ and IgM/hκ).

As healthy B cell development is essential for immunoglobulin production and effectively mounting humoral immune responses to immunization, we first investigated the effect of b*IGLJ-IGLC* gene cluster deletion on B cell development in the five newborn cKSL-HACΔ/TKO calves. For comparison, we also analyzed B cell development in newborn cKSL-HACΔ/DKO calves that we reported previously (Sano *et al.,* 2013). Flow cytometry analysis demonstrated that the percentages of hIgM/hIgκ (or hIgM/bIgκ)-double positive B cells slightly increased in cKSL-HACΔ/TKO Tc cattle over the DKO Tc cattle (Figure 6). On the contrary, no detectable levels of hIgG/hIgλ-double positive B cells were found, indicating that hIgκ is the predominant human light-chain expressed in these Tc cattle (data not shown). We found that the percentage of hIgM-single positive and hIgM/CD21-double positive B cells in cKSL-HACΔ/TKO Tc cattle were lower than those in cKSL-HACΔ/DKO (Figure 6).

**Figure 7:**
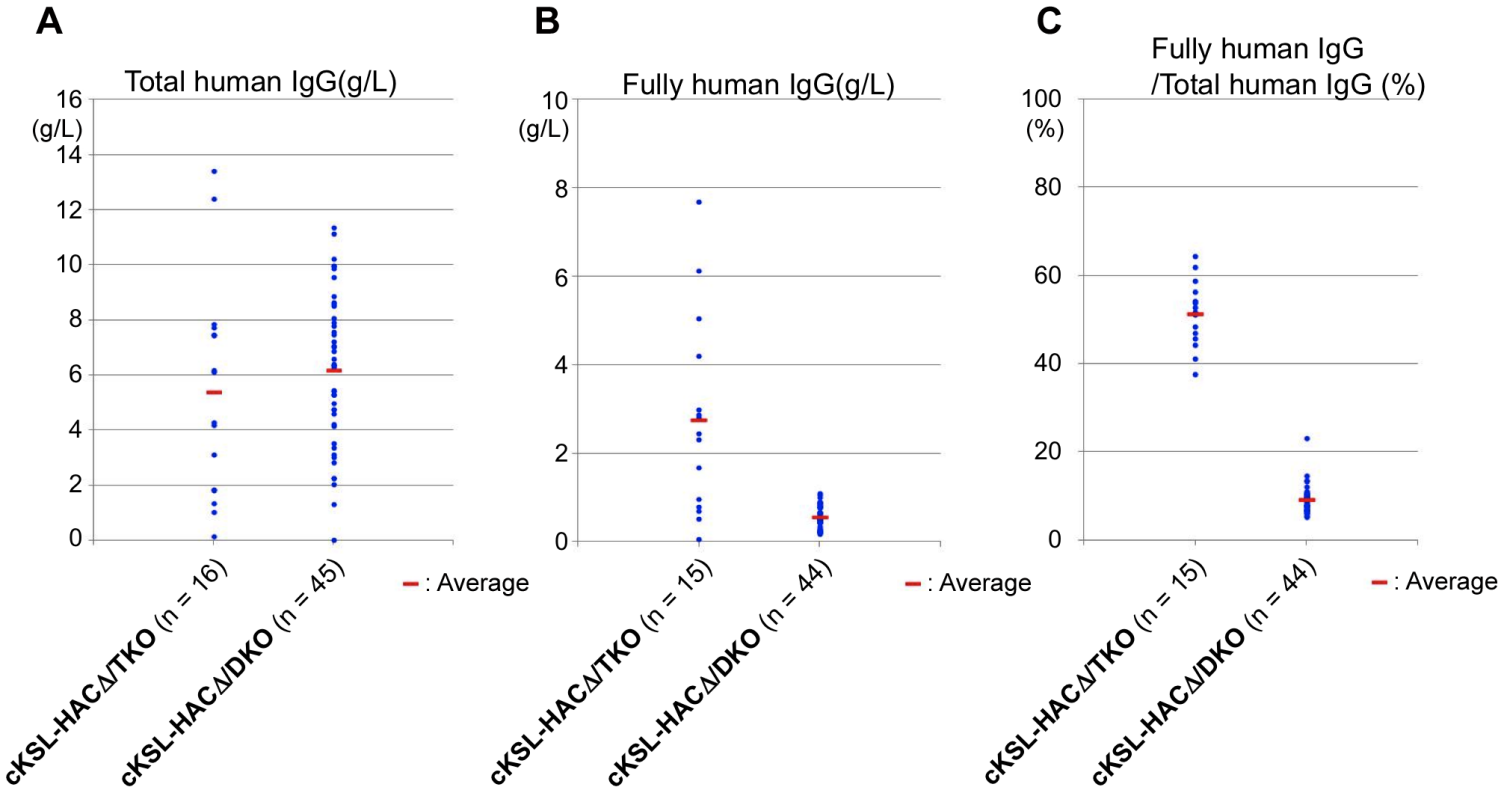
Serum concentrations of (A) total hIgG (g/l), (B) fully hIgG (hIgG/hκ plus hIgG/hIgλ) (g/l) and (C) serum fully hIgG over total hIgG (%) in cKSLHACΔ/TKO and cKSLHACΔ/DKO calves at 5-6 months of age. n = number of animals analyzed for each genotype. For each genotype, values of each measurement were plotted in each graph as blue closed circles. Red short lines represent arithmetic mean for each genotype in total hIgG (g/l), fully hIgG (hIgG/hκ plus hIgG/hIgλ) (g/l), and serum fully hIgG (hIgG/hκ plus hIgG/hIgλ) over total hIgG (%).

When the Tc calves reached 5-6 months of age, we measured the serum concentrations of total hIgG and fully hIgG in the cKSL-HACΔ/TKO Tc calves and compared them to those in age-matched cKSL-HACΔ/DKO Tc calves. As shown in Figure 7, the average levels of total hIgG were comparable or slightly lower in the sera of cKSL-HACΔ/TKO Tc calves than those from cKSL-HACΔ/DKO Tc calves (Figure 7). More importantly, the percentages of fully hIgG drastically increased in TKO Tc calves over the DKO Tc calves (Figure 7). Therefore, we achieved our goal of significantly improving fully hIgG production by inactivating the b*IGL* locus, resulting in, on average, about 51.3% of the total hIgG as fully hIgG (Figure 7).

## Discussion

Due to the overwhelming demands for human plasma derived hIgGs to treat a variety of human disease, its supply has been in a severe shortage [1], [8]. To address this problem, IgGs produced from hyper-immunized animals have been used as an alternative for treating certain human diseases for which it may be effective. One example is the use of IgGs prepared from plasma of immunized horses or sheep to treat severe envenomation resulting from snake and spider bites [9], [10]. While large quantities of IgGs can be produced from animals at relative low costs, animal derived IgGs are immunogenic in humans causing serum sickness, which not only reduces the efficacy of the IgGs but also places treated patients at risk of developing complications [11], [12].

We previously generated Tc cattle in which both of the two bovine Ig mu heavy-chain loci were homozygously inactivated by gene targeting (b*IGHM* ^−*/*−^ and b*IGHML1* ^−*/*−^) and the bovine immunoglobulin gene function was reconstituted by a HAC comprising the entire unrearranged germline loci of human immunoglobulin genes (Sano A *et al*., 2013). We demonstrated that DKO Tc cattle produce physiological level of hIgGs. Furthermore, through immunization studies these Tc cattle were shown to be responsive to antigen challenge generating high titers and functional antigen-specific hIgGs. However, as the bovine endogenous immunoglobulin light-chain genes are intact in these Tc cattle and the bovine genes may be epigenetically favored for expression in bovine B cells (e.g. regulated by bovine trans-elements) over the human immunoglobulin light-chain genes carried by the HAC, the majority of hIgGs produced from these cattle were chimeric, with the IgG heavy-chain of human origin and light-chain of bovine origin (Sano A et al., 2013).

Obviously, further genetic inactivation of the bovine Ig light-chain genes would increase fully human IgG production in these Tc cattle. It has been well established that, between the two bovine Ig light-chain loci, b*IGL* and b*IGK*, the b*IGL* locus is the predominant bovine Ig light-chain over the b*IGK* locus, resulting in an Igλ/Igκ ratio of 91/9 in wild type cattle [13]. Therefore, as the next step towards fully hIgG production in Tc cattle, we chose to genetically inactivate the bovine b*IGL* locus. However, as there are about 25 lambda variable (*IGLV*) genes spanning about 400 kb in the bovine genome [7], neither an individual KO nor a whole IGLV cluster deletion seemed to be a feasible approach. This is particularly true considering the fact that there are no precedent studies showing that the Cre/loxP strategy is effective in deleting large chromosomal fragments in somatic cells. Consequently, we decided to inactivate the b*IGL* locus by deleting the *IGLJ-IGLC* gene cluster, as the size has been previously estimated to be approximately 18.6 kb (Chen L *et al*. 2008; our more detailed sequence analysis indicates it is about 27 kb; see results section of this manuscript). We reasoned that the relative smaller size of this locus compared to the *IGLV* locus could be more amenable to Cre/loxP-mediated deletion. Therefore, we conducted detailed characterization of the bovine *IGLJ-IGLC* gene cluster, which allowed us to design and construct the gene targeting vectors to completely delete this locus. We demonstrated that the Cre/loxP system was very effective in inducing chromosome deletions in a site-specific manner in primary somatic cells. To the best of our knowledge, this was the first demonstration of this application of the Cre/loxP system in cultured primary somatic cells in any mammalian species.

Apart from the challenges to conduct large DNA fragment deletions in the chromosomes of primary somatic cells, modification of multiple genomic loci in primary somatic cells is prohibitive due to the limited life span of somatic cells in cell culture. Previously, we developed a sequential gene targeting strategy and achieved inactivation of up to six genomic loci in bovine somatic cells. However, if this sequential gene targeting strategy were followed in the instance of knocking out the *IGLJ-IGLC* gene cluster, it would take ten rounds of gene targeting and embryonic cloning to homozygously inactivate the two *IGH* loci and delete the *IGLJ-IGLC* gene cluster—four rounds of gene targeting and embryonic cloning would be needed to inactivate the four alleles of the *IGH* genes and six more rounds would be needed to homozygously delete the *IGLJ-IGLC* gene cluster (deleting one allele requires two rounds of gene targeting and embryonic cloning to integrate the two loxP sequences at the 5′ and 3′ of the *IGLJ-IGLC* gene cluster, respectively, and the induction of *IGLJ-IGLC* gene cluster deletion by transfecting cells with Cre expression vector requires another round of gene targeting and embryonic cloning; the same applies for the second allele also). To reduce the rounds of embryonic cloning and avoid the cumulative epigenetic errors that are introduced at each round of embryonic cloning, which can severely compromise the development potential of embryos reconstituted from the genetically modified cells by CT (our unpublished data), we decided to hemizygously delete the *IGLJ-IGLC* gene cluster in both a male and a female bovine cell line in which the *IGHM* and *IGHML* loci had been modified to produce male and female animals and breed them to produce homozygous offspring. To reduce the rounds of embryonic cloning even further, after integrating the first loxP sequence at 5′ of the *IGLJ1* gene, we co-transfected the second loxP KI vector with the Cre expression vector to achieve integration of the second loxP sequence and the subsequent deletion of the *IGLJ-IGLC* cluster between the two integrated loxP sequences with one round of cell transfection. We demonstrated that this strategy was very successful and allowed us to achieve the goal of producing Tc calves with both of the *IGHM* and *IGHML* loci and the *IGL* locus homozygous inactivated (*IGHM* ^−/−^ *IGHML1* ^−/−^ *IGL* ^−/−^).

As expected, homozygous inactivation of the b*IGL* locus through deleting the *IGLJ-IGLC* gene cluster resulted in dramatically increased serum concentration of fully hIgG as compared to the DKO/Tc cattle, with an average of 2.8 g/l. To the best of our knowledge, this is the highest fully hIgG production in any transgenic animal systems reported so far [14]. Therefore, through an unprecedented genome engineering effort in cattle, we succeeded in the establishment of a Tc bovine platform that is capable of efficiently producing fully hIgGs. We believe that this study is of great significance as this Tc bovine system has, for the first time, made using genetically engineered animals for large quantities of fully hIgGs production a reality towards providing a safe and reliable alternative to human plasma derived hIgG for human therapy.

Nevertheless, while the work presented here is a great step toward establishing the Tc bovine platform for fully human antibody production, our final goal has yet to be fully accomplished: the bovine Ig kappa light-chain locus, b*IGK*, is still intact in the TKO/Tc cattle, resulting in the production of chimeric human antibodies composing of human Ig heavy-chains and bovine Ig kappa chains. Therefore, we are working to homozygously inactivate the b*IGK* locus through gene targeting to produce quadruple bovine Ig gene KO (QKO) Tc cattle. We envision that the creation of QKO/Tc cattle will eliminate the need to purify fully human antibodies from the chimeric human antibodies. In addition, as an effort to improve fully human antibody production by Tc bovine, we have recently achieved the bovinization of some sequences on the HAC that are important for Ig function and/or expression, resulting in an average of over 80% of the serum IgGs produced from the TKO/Tc cattle to be fully human IgGs (manuscript in preparation).

Because our ultimate goal in engineering a Tc bovine system is to produce fully human therapeutic antibodies, we have also conducted studies to produce and functionally characterize antigen-specific fully human antibodies from hyper-immunized Tc cattle against a panel of human diseases, such as viral and cancer antigens. Such research includes the development of proprietary immunogen formulation and immunization protocols to hyper-immunize the Tc cattle, the development of cost-effective antibody purification procedures even with current fully human antibody levels. We have demonstrated, as a proof of concept, that pathogen-specific fully human antibodies produced from hyper-immunized Tc cattle are highly effective in neutralizing target antigens both *in vitro* and *in vivo*. Currently, we are preparing for the first clinical trial studies for pathogen-specific fully human antibodies derived from Tc bovine.

## Materials and Methods

### Ethics statement

The animal protocols contained in the study were approved by the Hematech (previous name of Sanford Applied Biosciences) Institutional Animal Care and Use Committee (IACUC) (USDA Research Facility 46-R-0008, OLAW #A4438-01, AAALAC #001114). Care of all vertebrate animals was subject to regular review by the IACUC and complied with Animal Welfare laws and regulations of the United States. Periodic health evaluations (blood profile, weight, etc.) were made by Veterinary Service to ensure that Tc cattle were healthy. All Tc bovine received adequate housing, feed, access to water and bedding. Daily observations were made by herdsmen to ensure that appropriate standards of animal care were being met. Sanford Applied Biosciences uses the *Guide for the Care and Use of Laboratory Animals* and the *Guide for the Care and Use of Agricultural Animals Used in Agricultural and Research Teaching* for animal care standards. Animal care and use and facilities were inspected by the IACUC on a semiannual basis. Animals may have to be euthanized due to unfortunate events like terminal illness and trauma. Methods of euthanasia followed the guidelines given by the *American Veterinary Medical Association (AVMA) 2013*. These are acceptable methods by the *AVMA* and they were approved by the IACUC. All protocols and procedures used in the Animal Care and Use Program that may have caused discomfort, distress and pain, and injury were reviewed by the IACUC for appropriate management practices. Analgesic, anesthetics and tranquillizing drugs were used when appropriate under supervision of Veterinary Services. Veterinarians and herdsmen used cattle chute for restraining when needed. Cattle were not restrained for more than four hours per day.

### BAC genomic library

The bovine genomic BAC library (CHORI-240) was purchased from Children's Hospital Oakland Research Institute and screening was performed, according to their instruction.

### Shotgun sequencing of BAC

Shotgun sequencing of a BAC clone (141C2) was performed by Shimadzu Genomic Research (Kyoto, Japan).

### Southern blotting

Approximately 20 µg genomic DNA digested with BamHI, Bgl II, EcoRI, HindIII and Pst I, respectively, were separated by agarose gel electrophoresis. The separated DNA were subsequently transferred to positively charged nylon membrane (Hybond N+, Amersham) after depurination with 0.25N HCl for 15 minutes twice, neutralization in 0.5N NaOH/1.5M NaCl solution for 15 minutes twice, washed in 1M Tris-Cl (pH7.0)/1.5M NaCl solution for 20 minutes twice and rinsed with 0.2x SSC once. After DNA transfer, the membrane was rinsed with 0.2xSSC, air-dried, UV cross-linked, and pre-hybridized in 5xDenhards's with 100 µg/ml of single stranded DNA for 3 hours at 65°C. Probe was prepared with Rediprime II labeling kit (Amersham) using 25 ng of 200 bp bovine Cλ cDNA fragment following manufacturer's instructions. After pre-hybridization was completed, the membrane was hybridized with the probe over night at 65°C and washed by following manufacturer's instruction. A film (Kodak Safety Film AR, Kodak) was exposed for 5 days with an intensifying screen at −80°C.

### Construction of gene targeting vectors

#### Gene targeting vectors for knocking out the bIGH locus

The construction of gene targeting vectors, pbCμAYKObsr for *10AY*, pBCμΔNKOneo for *U*, and pBCμΔKOpuro for *u*, were described previously [5].

#### Construction of gene targeting vector pBCμ7AY^J^KOhyg for the 7AY^J^ allele

To isolate isogenic DNA from the *bIGH* locus for constructing the gene targeting vector, we constructed a lambda phage genomic library by using the genomic DNA isolated from a Holstein x Jersey cross primary fetal cell line (Lofstrand Labs, Gaithersburg, MD, USA). We screened the lambda phage library with ^32^P labeled BCμ-f2/BCμ-r2 PCR product as the probe and identified multiple positive phage clones. We then subcloned a Sal I fragment (about 17 kb) isolated from a lambda clone (#7) into the Sal I site of pBluescript II SK(-). From this plasmid, a 9.8 kb BamHI fragment was further cloned into the BamHI site of pBluescript II SK(-). To this resulting plasmid, we then inserted a STOPhyg cassette into the Bgl II site and DT-A cassette into Not I site to generate the pBCμ7AY^J^KOhyg gene targeting vector.

### Gene targeting vectors for bIGLJ-IGLC gene cluster deletion

#### Construction of gene targeting vector pCλ1CAGzeoPuro^loxP^DT

A 13 kb *Nde* I-*Hin* dIII genomic fragment isolated from a lambda phage clone was subcloned into pBluescript II SK(-) vector and the CAGzeo/loxP/promoter-less *puro* cassette was inserted at *Afe* I site present in the genomic fragment. Then the *DT-A* gene was inserted at *Not* I site. This vector was constructed from the alleles *A* and *B* genomic fragment derived from lambda phage clones.

#### Construction of gene targeting vector pCλ5CAG^loxP^neoDT

A 10 kb *Sac* II-*Nsi* I genomic fragment of a BAC clone positive for 3′ side of the *IGLJ5-IGLC5* gene was subcloned into pBluescript II SK(-)vector and the CAG promoter/loxP/polyA/*neo* cassette was inserted at *Hin* dIII site present in the genomic fragment. Finally, *DT-A* gene was inserted at *Not* I site. This vector was constructed from the alleles *A* and *B* genomic fragment derived from lambda phage clones.

### Transfection of bovine fibroblasts for the bovine *IGLJ-IGLC* gene cluster deletion and MMCT

Bovine fetal fibroblasts were cultured and transfected as previously described [5]. Briefly, fibroblasts were electroporated with ∼30 µg of each targeting vector at 550 V and 50 µF. After 48 hours, the cells were selected under an appropriate drug: zeocin (0.4 mg/ml) or puromycin (1 µg/ml) for two weeks, and resistant colonies were selected and transferred to replica plates; one for genomic DNA extraction and the other for embryonic cloning. MMCT was done with each HAC vector as described previously [5].

### Genomic PCR and RT-PCR analyses

These analyses were implemented as previously described [5]. All the PCR products were run on 0.8% agarose gels. Primer sequences are available from Supplementary information (Figure S4).

### Flow cytometry analysis

Flow cytometry analysis on B cell development in newborn Tc calves was performed as previously described (Kuroiwa Y, *et al.* 2004) with the following modifications. To detect surface hIgG on Tc bovine B cells, goat anti-hIgG (Life Technologies) directly labeled with AF 488 was used. To label surface hIgê on Tc bovine B cells, mouse anti-hIgê antibody (Biolegend) directly labeled with PE was used. To label surface bIgë or bIgê on the B cells, mouse monoclonal anti-bIgë (in-house clone 132D7) or mouse monoclonal anti-bIgê (in-house clone 132B10) followed by Zenon mouse IgG1PE labeling (Life Technologies) were used. Staining was done by a standard protocol and then analyzed by FACSAria flow cytometer (BD Biosciences).

### ELISA

Total hIgG ELISA assay was performed as previously described (Kuroiwa Y, *et al.* 2004). For hIgG/hIgê or hIgG/hIgλ detection, goat anti-hIgκ affinity-purified or goat anti-hIgλ affinity-purified (Bethyl) as a capture and goat anti-hIgG Fc-HRP (Bethyl) as a detection antibody were used.

### Animal cloning by somatic cell chromatin transfer

Cloned fetuses and calves were produced using chromatin transfer procedure described previously (Sullivan, E *et al*. 2004).

## Supporting Information

Figure S1 **DNA sequence alignment between the bovine** ***IGLJ2-IGLC2*** **and** ***IGLJ3-IGLC3*** **genes.** (A) Intron DNA sequence alignment between the bovine *IGLJ2-IGLC2* and *IGLJ3-IGLC3* genes. “JL2-CL2” and “JL3-CL3” corresponds to intronic sequence of the *IGLJ2-IGLC2* and *IGLJ3-IGLC3* genes, respectively. (*B*) 3′UTR (untranslated region) DNA sequence alignment between the bovine *IGLC2* and *IGLC3* genes. “bCL2” and “bCL3” corresponds to 3′UTR sequence of the *IGLC2* and *IGLC3* genes, respectively.
(TIF)

Figure S2 **The origin of the five TKO cell lines is shown.** All TKO cell lines originated from Holstein male cell line 6939 and Holstein-Jersey cross bred female cell line 3427. 6939 was sequentially targeted three times to generate *IGM^−/+^IGML1^−/−^*calf 305(G3). 3427 was sequentially targeted two times to generate *IGM^−/−^* calves 1295, 1296, 1305. The male calf 305 and female calves 1295, 1296 and 1305 were used for the 1^st^ breeding to obtain IGM*^−^* ^/*−*^ IGML1*^−^* ^/+^ male (J481) and female (H412 and J746) fetal cell lines. These fetal cell lines were further sequentially targeted two times with Cre-loxP recombination for the *IGLJ-IGLC* cluster deletion at the same time as the second targeting. Resulting male *IGM^−/−^IGML1^−/+^IGL^−/+^* calves 522, 526 and 528, and the female *IGM^−/−^IGML1^−/+^IGL^−/+^* calves 1768, 1804, 1805, 1806 and 1820 were used for the second breeding to generate five TKO fetal cell lines, E024A-2, A332A, A596A-1, A114A and C970A.
(TIF)

Figure S3 **The complete 141C2 BAC sequence is shown. Bovine IGLJ1-IGLC1 is located at 9480-9517 and 10791-11110.** Bovine IGLJ2-IGLC2 is located at 15707-15744 and 17048-17367. Bovine IGLJ3-IGLC3 is located at 22023-22060 and 23364-23683. Bovine IGLJ4-IGLC4 is located at 28820-28857 and 30153-30472. Bovine IGLJ5-IGLC5 is located at 35097-35134 and 36422-36740. These sequence regions are underlined in red.
(GBK)

Figure S4 **List of primer sequences for genomic PCR and RT-PCR used for analysis of bovine immunoglobulins.**
(XLSX)

## References

[1] Kumar A, Teuber SS, Gershwin ME (2006) Intravenous immunoglobulin: striving for appropriate use. Int Arch Allergy Immunol 140: 185–198. 16682800 10.1159/000093204

[2] Yeh KM, Chiueh TS, Siu LK, Lin JC, Chan PK, et al (2005) Experience of using convalescent plasma for severe acute respiratory syndrome among healthcare workers in a Taiwan hospital. J Antimicrob Chemother 56: 919–922. 16183666 10.1093/jac/dki346 PMC7110092

[3] Kuroiwa Y, Kasinathan P, Matsushita H, Sathiyaselan J, Sullivan EJ, et al (2004) Sequential targeting of the genes encoding immunoglobulin-mu and prion protein in cattle. Nat Genet 36: 775–780. 15184897 10.1038/ng1373

[4] Sullivan EJ, Kasinathan S, Kasinathan P, Robl JM, Collas P (2004) Cloned calves from chromatin remodeled in vitro. Biol Reprod 70: 146–153. 13679310 10.1095/biolreprod.103.021220

[5] Kuroiwa Y, Kasinathan P, Sathiyaseelan T, Jiao JA, Matsushita H, et al (2009) Antigen-specific human polyclonal antibodies from hyperimmunized cattle. Nat Biotechnol 27: 173–181. 19151699 10.1038/nbt.1521

[6] Chen L, Li M, Li Q, Yang X, An X, et al (2008) Characterization of the bovine immunoglobulin lambda light chain constant IGLC genes. Vet Immunol Immunopathol 124: 284–294. 18538861 10.1016/j.vetimm.2008.04.012

[7] Pasman Y, Saini SS, Smith E, Kaushik AK (2010) Organization and genomic complexity of bovine lambda-light chain gene locus. Vet Immunol Immunopathol 135: 306–313. 20171743 10.1016/j.vetimm.2009.12.012

[8] Buchacher A, Iberer G (2006) Purification of intravenous immunoglobulin G from human plasma—aspects of yield and virus safety. Biotechnol J 1: 148–163. 16892245 10.1002/biot.200500037

[9] Newcombe C, Newcombe AR (2007) Antibody production: polyclonal-derived biotherapeutics. J Chromatogr B Analyt Technol Biomed Life Sci 848: 2–7. 10.1016/j.jchromb.2006.07.004 16893686

[10] Sellahewa KH, Kumararatne MP, Dassanayake PB, Wijesundera A (1994) Intravenous immunoglobulin in the treatment of snake bite envenoming: a pilot study. Ceylon Med J 39: 173–175. 7728916

[11] Boothpur R, Hardinger KL, Skelton RM, Lluka B, Koch MJ, et al (2010) Serum sickness after treatment with rabbit antithymocyte globulin in kidney transplant recipients with previous rabbit exposure. Am J Kidney Dis 55: 141–143. 19628314 10.1053/j.ajkd.2009.06.017 PMC2803326

[12] Lundquist AL, Chari RS, Wood JH, Miller GG, Schaefer HM, et al (2007) Serum sickness following rabbit antithymocyte-globulin induction in a liver transplant recipient: case report and literature review. Liver Transpl 13: 647–650. 17377915 10.1002/lt.21098

[13] Arun SS, Breuer W, Hermanns W (1996) Immunohistochemical examination of light-chain expression (lambda/kappa ratio) in canine, feline, equine, bovine and porcine plasma cells. Zentralbl Veterinarmed A 43: 573–576. 8968166 10.1111/j.1439-0442.1996.tb00489.x

[14] Tomizuka K, Shinohara T, Yoshida H, Uejima H, Ohguma A, et al (2000) Double trans-chromosomic mice: maintenance of two individual human chromosome fragments containing Ig heavy and kappa loci and expression of fully human antibodies. Proc Natl Acad Sci U S A 97: 722–727. 10639146 10.1073/pnas.97.2.722 PMC15397

