# Correction: Triple Immunoglobulin Gene Knockout Transchromosomic Cattle: Bovine Lambda Cluster Deletion and Its Effect on Fully Human Polyclonal Antibody Production

**DOI:** 10.1371/journal.pone.0099279

**Published:** 2014-05-27

**Authors:** The PLOS ONE Staff

Three references are omitted from the References list, but are cited in the text of the article. The authors have provided the references here:

Hilgenfeld R, Peiris M (2013) From SARS to MERS: 10 years of research on highly pathogenic human coronaviruses**.** Antiviral Res 100(1): 286-295. doi: 10.1016/j.antiviral.2013.08.015. [Epub ahead of print]

Hosseini A, Campbell G, Prorocic M, Aitken R (2004) Duplicated copies of the bovine JH locus contribute to the Ig repertoire. Int Immunol 16: 843–852.

Sano A, Matsushita H, Wu H, Jiao J-A, Kasinathna P, Sullivan EJ, Wang Z, Kuroiwa Y (2013) Physiological Level Production of Antigen-Specific Human Immunoglobulin in Cloned Transgenic Cattle. PLoS ONE 8(10): e78119, doi:10.1371/journal.pone.0078119
