# Supplementary figures and images for: Triple Immunoglobulin Gene Knockout Transchromosomic Cattle: Bovine Lambda Cluster Deletion and Its Effect on Fully Human Polyclonal Antibody Production
Source: PLoS One. 2014 Mar 6;9(3):e90383. doi: 10.1371/journal.pone.0090383 (PMC3946162; doi:10.1371/journal.pone.0090383)

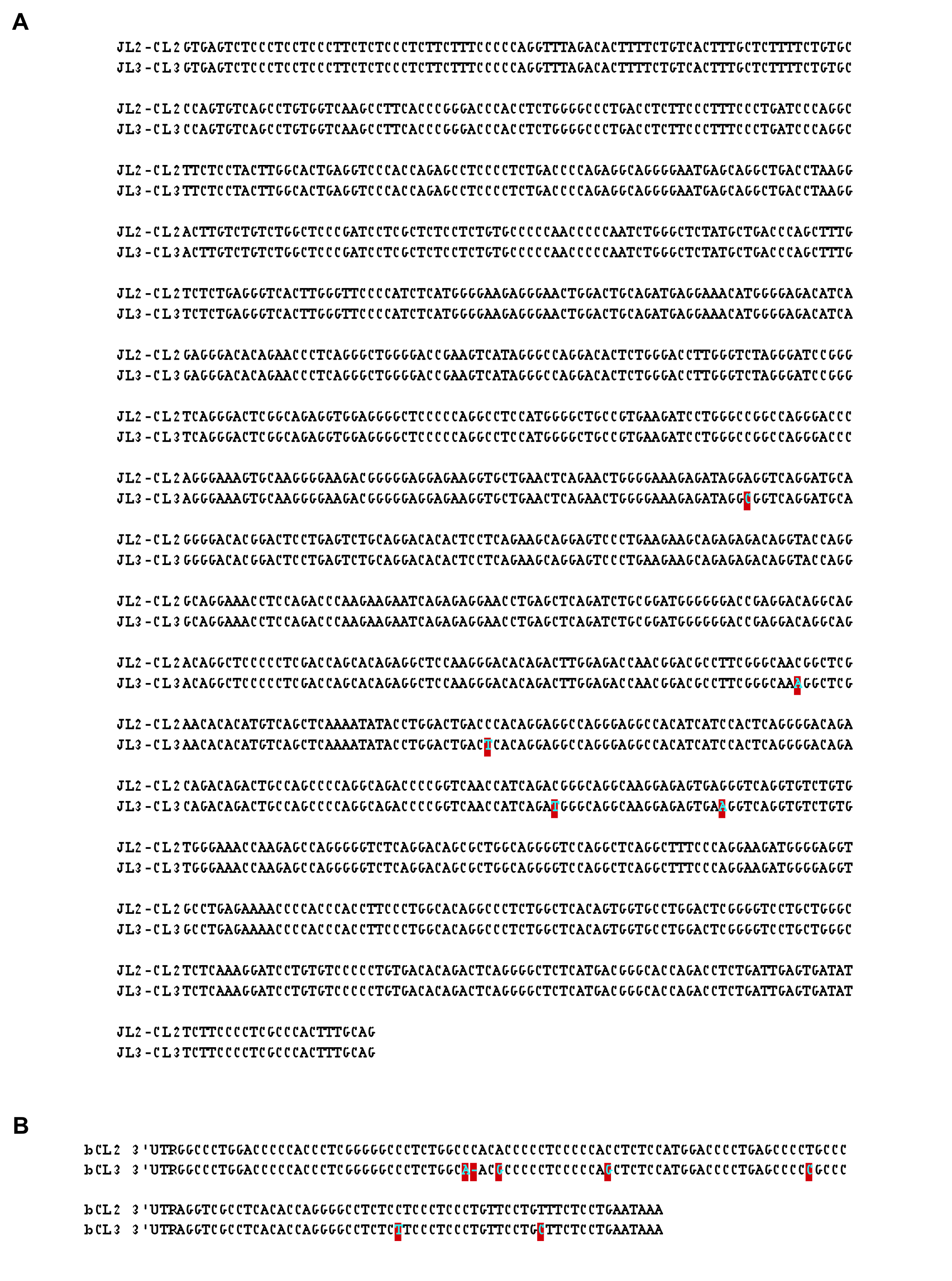

Supplement: Figure S1 — DNA sequence alignment between the bovine IGLJ2-IGLC2 and IGLJ3-IGLC3 genes. (A) Intron DNA sequence alignment between the bovine IGLJ2-IGLC2 and IGLJ3-IGLC3 genes. “JL2-CL2” and “JL3-CL3” corresponds to intronic sequence of the IGLJ2-IGLC2 and IGLJ3-IGLC3 genes, respectively. (B) 3′UTR (untranslated region) DNA sequence alignment between the bovine IGLC2 and IGLC3 genes. “bCL2” and “bCL3” corresponds to 3′UTR sequence of the IGLC2 and IGLC3 genes, respectively. (TIF) [file pone.0090383.s001.tif]

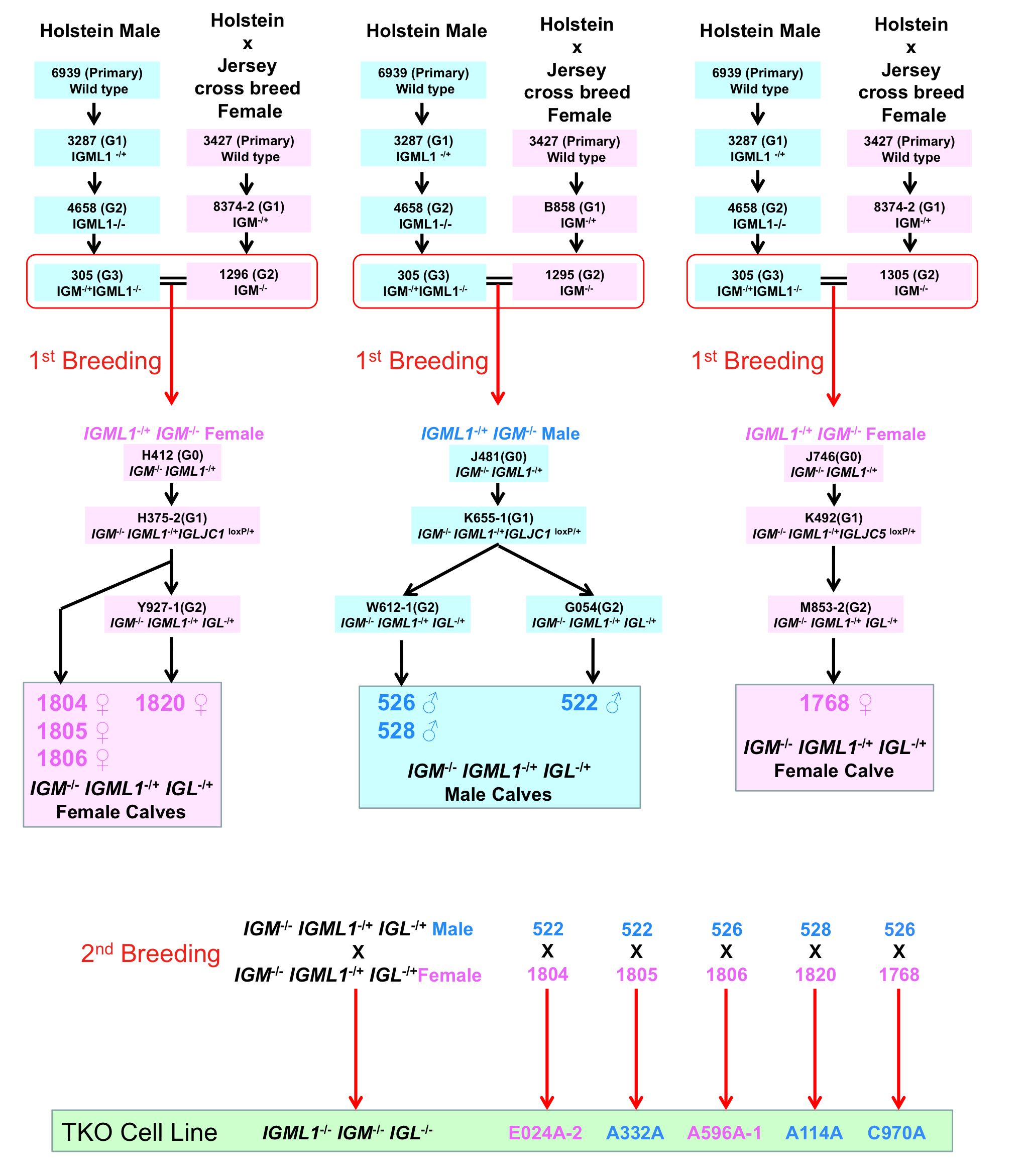

Supplement: Figure S2 — The origin of the five TKO cell lines is shown. All TKO cell lines originated from Holstein male cell line 6939 and Holstein-Jersey cross bred female cell line 3427. 6939 was sequentially targeted three times to generate IGM−/+IGML1−/−calf 305(G3). 3427 was sequentially targeted two times to generate IGM−/− calves 1295, 1296, 1305. The male calf 305 and female calves 1295, 1296 and 1305 were used for the 1st breeding to obtain IGM− /− IGML1− /+ male (J481) and female (H412 and J746) fetal cell lines. These fetal cell lines were further sequentially targeted two times with Cre-loxP recombination for the IGLJ-IGLC cluster deletion at the same time as the second targeting. Resulting male IGM−/−IGML1−/+IGL−/+ calves 522, 526 and 528, and the female IGM−/−IGML1−/+IGL−/+ calves 1768, 1804, 1805, 1806 and 1820 were used for the second breeding to generate five TKO fetal cell lines, E024A-2, A332A, A596A-1, A114A and C970A. (TIF) [file pone.0090383.s002.tif]
